# Effects of Warming and Phosphorus Enrichment on the C:N:P Stoichiometry of *Potamogeton crispus* Organs

**DOI:** 10.3389/fpls.2022.814255

**Published:** 2022-03-29

**Authors:** Mingzhe Dai, Tao Wang, Yuyu Wang, Jun Xu

**Affiliations:** ^1^School of Ecology and Nature Conservation, Beijing Forestry University, Beijing, China; ^2^Donghu Experimental Station of Lake Ecosystems, State Key Laboratory of Freshwater Ecology and Biotechnology of China, Institute of Hydrobiology, Chinese Academy of Sciences, Wuhan, China; ^3^College of Advanced Agricultural Sciences, University of Chinese Academy of Sciences, Beijing, China

**Keywords:** stoichiometric characteristics, eutrophication, climate change, growth organs, reproductive organs

## Abstract

The loss of submerged macrophytes from freshwater ecosystems is accelerating owing to the combined effects of eutrophication and climate change. Submerged macrophytes depend on spring clear water; however, increased water temperatures and excessive phosphorus (P) inputs often lead to the dominance of phytoplankton. It is still not clear how the stoichiometric characteristics of carbon (C), nitrogen (N), and P in different tissues of submerged macrophytes respond to P enrichment and temperature increases. In this study, we established 36 mesocosm ecosystems to explore the effects of warming and P addition on the leaf, turion, stem, and seed stoichiometry of *Potamogeton crispus*. The results revealed that different functional plant organs show distinct responses to P addition and warming, which demonstrates the importance of evaluating the responses of different submerged macrophyte organs to environmental changes. In addition, interactive effects between P addition and warming were observed in the leaf, turion, and seed C:N:P stoichiometry, which highlights the importance of multifactorial studies. Our data showed that warming caused a decrease in the C content in most organs, with the exception of the stem; P addition increased the P content in most organs, with the exception of seed; N content in the turion and seed were influenced by interactive effects. Collectively, P addition could help *P. crispus* to resist the adverse effects of high temperatures by aiding growth and asexual reproduction, and asexual propagules were found to be more sensitive to P enrichment than sexual propagules.

## Introduction

Submerged macrophytes are the primary producers in lakes and play an important role in maintaining clean water (Su et al., 2017). However, globally, the loss of submerged aquatic vegetation is accelerating. A meta-analysis of 155 lakes found that, in 65.2% of the lakes, the aquatic vegetation cover decreased (Zhang et al., 2017). Eutrophication (Sondergaard et al., 2010) and climate change (Phillips et al., 2016) are the two main causes of the decline and disappearance of submerged macrophytes. Currently, eutrophication is accelerating, and eutrophication in lakes in mid- and low-latitude regions is more serious than that in lakes in high-latitude regions (Izmailova and Rumyantsev, 2016). The shallow lakes in the middle-lower reaches of the Yangtze River suffer from high-nutrient input from both inflows and sediments (Jin et al., 2005; Le et al., 2010; Qin et al., 2020). Shallow lakes with a large surface area to volume ratio and no thermal stratification are more sensitive to climate warming than other types of lakes (Gerten and Adrian, 2000; Mooij et al., 2005). The frequency and intensity of extreme climate events (such as heatwaves) are expected to increase over the next 100 years (Ferreira et al., 2010; Perkins et al., 2012). A regional model predicts that temperature will increase by 4.9 ± 0.95°C (RCP 8.5) over southern China by the century’s end, compared with the temperature from 1980 to 1999 (Chen and Frauenfeld, 2014a). Furthermore, the duration and intensity of heatwaves are predicted to increase significantly in the middle and lower reaches of the Yangtze River (Qi and Yang, 2019).

Carbon (C) is the most essential element that constitutes the dry matter in plants; nitrogen (N) determines plant growth by regulating the number and size of organs, and balancing nutrition and reproductive growth, whereas phosphorus (P) influences leaf formation and shape, as well as the plant’s flowering and seed formation (Plénet et al., 2000; Marschner, 2012). The N/C and P/C ratios determine the relative growth rate of plants (Ågren, 2004), and the N/P ratio reflects the restriction of plant growth (Wang et al., 2014). During plant growth, the C:N:P stoichiometry responds to environmental conditions (Zhang et al., 2013) and is related to important ecological processes, such as N_2_ fixation (Sañudo-Wilhelmy et al., 2001), litter decomposition (Güsewell and Gessner, 2009), species diversity (Ren et al., 2017; Chen and Chen, 2021), and the ability of organisms to adapt to environmental stress (Xie et al., 2018). Plant stoichiometry varies with the growth rate and the surrounding environment. Control experiments have shown that rising temperatures affect the growth, stoichiometry, and palatability of submerged macrophytes (Short et al., 2016; Zhang et al., 2019; Xu et al., 2020). Elevated temperatures lead to a decrease in N and P contents in aquatic plants, which results in an increase in C/N and C/P ratios. A study on terrestrial plants and phytoplankton showed that this effect was caused by an increase in nutrient use efficiency (An et al., 2005; De Senerpont Domis et al., 2014). The increase in temperature also changes the diffusion rate of nutrients in the water, owing to changes in the boundary layer surrounding organisms, which leads to changes in the stoichiometry of organisms (Raven and Geider, 1988). Studies have shown that warming may reduce the C/N ratio of terrestrial plants by increasing plant productivity, biological activity, and nutrient absorption (Welker et al., 2005; Aerts et al., 2009). However, in temperate terrestrial ecosystems, plant C/N and C/P ratios may increase (Sardans et al., 2012). The recognized importance of P as a limiting factor in terrestrial and aquatic ecosystems is increasing (Peñuelas et al., 2012). Nutrient addition can positively impact the nutritional quality of aquatic plants (Burkholder et al., 2007; Sardans et al., 2012; Bakker and Nolet, 2014). However, eutrophication can reduce the C/P ratio of algae in rivers (Frost and Elser, 2002; Dang et al., 2009), and low C/P ratios yield greater advantages to fast-growing species and disadvantages to slow-growing taxa (Frost and Elser, 2002), which may reduce ecosystem biodiversity (Evans-White et al., 2009). In addition, changes in the N/P ratio often favor algal species that can compete for restricted nutrients, which gives them a potential advantage (Granéli et al., 2008) and causes problems for the restoration of submerged macrophytes. In summary, these studies have found that temperature rise and nutrient enrichment have opposing effects on the stoichiometric characteristics of aquatic organisms. Temperature rise is expected to increase the ratio, whereas nutrient enrichment is expected to decrease the ratio. However, there are few reports about how the stoichiometry of submerged macrophytes responds to external environment change (Güsewell, 2004).

*Potamogeton crispus* is a perennial aquatic plant and the dominant species in freshwater areas of East Asia (Kunii, 1982; Xie et al., 2003). In particular, it is one of the few species that survives in nutrient-rich Chinese lakes (Lu et al., 2012). It has a strong resistance to stress brought on by pollution, and its ability to absorb nutrients is often used in wetland restoration (Jin et al., 1994; Xu et al., 2015). In the subtropical and temperate regions of the northern hemisphere, the life cycle of *P. crispus* differs from that of most submerged macrophytes. The optimum temperature range for *P. crispus* is 10–20°C, thus its biomass and productivity peak in the spring (Kunii, 1982). In summer, the leaves and stems rot and die, and propagule turions and seeds fall into the water body. *P. crispus* can reproduce either asexually or sexually (Shen et al., 2008). Turions formed at the top of the stem are important for the asexual reproduction of *P. crispus*. Seeds are produced at the same time as turions are formed (Sastroutomo et al., 1979; Rogers and Breen, 1980; Kunii, 1982), but the germination rate of *P. crispus* seeds is low under natural conditions. Therefore, *P. crispus* relies mainly on asexual reproduction, especially turion reproduction (Chen et al., 2006). *P. crispus* can grow well in water with a total nitrogen concentration of less than 2 mg/L and a total phosphorus concentration of less than 0.4 mg/L (Ma, 2007), but it cannot survive nutrient concentrations that are very high (Zhang, 2006). Currently, few studies have explored how nutrient increase and global warming, and their interactive effects, impact the C:N:P stoichiometric ratios of the different organs associated with plant growth and reproduction.

In this study, we used outdoor mesocosms to investigate the effects of warming and P enrichment on the C:N:P stoichiometric relationship within the organs of *P. crispus*. Our hypotheses are as follows: (1) the contents of C, N, and P in the organs of *P. crispus* will increase with additional P input, but the stoichiometric ratio will decrease; (2) *P. crispus* is not tolerant of high temperatures; thus, the contents of C, N, and P in the organs will decrease with increasing temperature, thereby increasing the stoichiometric ratio; (3) the stoichiometric characteristics of *P. crispus’* organ responses vary under the interactive effects of warming and P input; and (4) seed stoichiometry is more stable than that of other organs because its function is to maintain the ability to reproduce in response to environmental changes.

## Materials and Methods

### Experiment Design

The outdoor mesocosm system was described in the study of Xu et al. (2020). The experiment was conducted from March 7 to May 30, 2018. Both water and plant samples were collected from Liangzi Lake. The treatments included two factors, temperature rise and P addition, with each treatment replicated six times. Tanks were randomly assigned to one of the six experiments, and the six treatments were divided into two categories: no phosphorus added and phosphorus added. The treatments were as follows: (1) no phosphorus added, which comprised natural water temperature with no added P (C); fixed heating with no added P (T), which comprised a constant increase in temperature that was 4°C above control conditions (in keeping with RCP 8.5 in the region by 2100) (Intergovernmental Panel on Climate Change (IPCC), 2013; Chen and Frauenfeld, 2014b); fluctuation of heating with no added P (V), where random temperature fluctuations between 0 and 8°C were applied based on the fixed heating group T. The total amount of warming over the duration of the experiment was equal for both temperature treatments (T and V). (2) Phosphorus added, which entailed P addition without warming (C + P) composed of the addition of 25 μg/L of KH_2_PO_4_ to the water every 2 weeks; fixed heating treatment and the P addition group (T + P), which comprised a constant increase in temperature that was 4°C above control conditions and the addition of 25 μg/L KH_2_PO_4_ every 2 weeks; fluctuation of heating and P addition (V + P), which had the same temperature setting as treatment V and had 25 μg/L KH_2_PO_4_ added every 2 weeks. The heating treatment began on the first day.

### Sampling and Analysis

Water samples were collected from each tank using a Plexiglas tube (diameter 70 mm; length 1 m) and then taken to the laboratory to measure the total nitrogen (TN), total phosphorus (TP), and Chl-*a* in the water according to the standards of the Chinese water analysis methods (Wang et al., 2008). An ultraviolet spectrophotometer (Cleverchem380, DeChem-Tech., Germany) was used to calculate the TP and TN weekly. The chlorophyll-*a* concentration was determined by filtering 500 ml of the water using Whatman GF/C filters and then using a spectrophotometer (UV-2800, Unico, China) after ethanol extraction (Supplementary Table 1). *P crispus* stems and leaves were collected every month from each tank, whereas the turions and seeds were collected at the end of the experiment to analyze the C, N, and P contents. The plant samples were dried in an oven at 70°C for 48 h to a constant weight, after which the dried samples were ground into powder using a ball mill (Mini Beadbeater-16, Biospec product, United States). The C and N contents were analyzed using an elemental analyzer (FlashEA1112, CE instrument, Italy) (Bradshaw et al., 2012; Lü et al., 2012). The P content was analyzed *via* digested plant samples with H_2_SO_4_-H_2_O_2_ and molybdenum antimony spectrophotometry (Kuo, 1996).

### Statistical Analysis

Two-way ANOVA was used to explore the effects of warming, P addition, and those of their interaction, on the TP, C/N, N/P, and C/P ratios of *P. crispus* turions, seeds, leaves, and stems using IBM SPSS Statistics 25 (SPSS, Chicago, Illinois, United States). The figures were drawn using Origin 2018 software (Origin Lab Corp., Massachusetts, United States).

## Results

### Effect of Warming and P Addition on the C, N, and P Contents and Stoichiometric Characteristics of *Potamogeton crispus* Leaves

**FIGURE 1:**
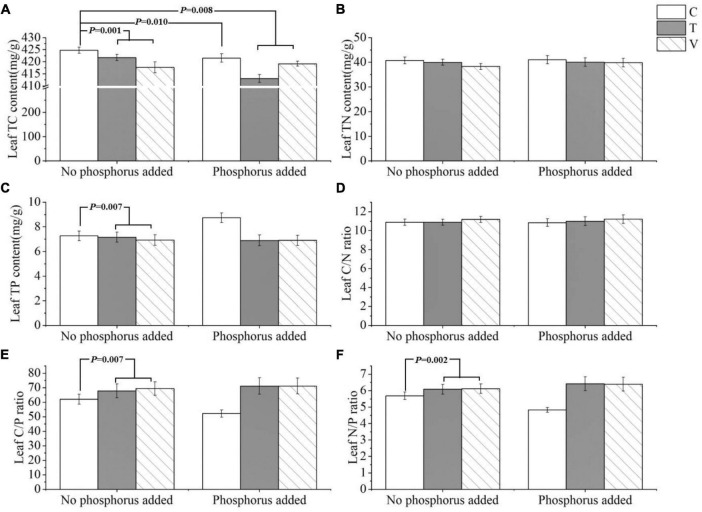
The stoichiometric characteristics of the **(A)** TC, **(B)** TN, and **(C)** TP contents and the **(D)** C/N, **(E)** C/P, and **(F)** N/P ratios of *P. crispus* leaves under different treatments. The C in “No phosphorus added” represent controls (C), T in “No phosphorus added” represent constant warming (T), and V in “No phosphorus added” represent variable warming (V). Meanwhile, the C in “Phosphorus Added” represent phosphorus addition (C + P), T + P in “Phosphorus Added” represent constant warming and phosphorus addition (T + P), and V + P in “Phosphorus Added” represent variable warming and phosphorus addition (V + P).

The leaf C content was significantly influenced by warming (*p* = 0.001), P addition (*p* = 0.01), and their interactive effects (*p* = 0.008). Leaf C content decreased when temperature increased, and it also decreased when P addition, warming, and warming and P addition interacted (Table 1 and Figure 1A). The N content and C/N ratio in the leaf showed no response (*p* > 0.05) (Table 1 and Figures 1B,D). The P content of the leaves was significantly affected by warming and it significantly decreased when temperature increased (*p* = 0.007) (Table 1 and Figure 1C). Furthermore, the C/P ratio (*p* = 0.007) and N/P ratio (*p* = 0.002) in the leaf were significantly affected by warming, and both significantly increased as temperature increased (Table 1 and Figures 1E,F).

**TABLE 1:** Effects of different temperature scenarios and phosphorus addition on the TC, TN, and TP contents and the C/N, C/P, and N/P ratios in *P. crispus* leaves.

Parameters	Factors	SS	df	MS	*F*	*p*-value
Leaf TC	T changes	1360.615	2	680.308	7.179	**0.001**
	P addition	645.826	1	645.826	6.815	**0.010**
	T changes *P addition	939.096	2	469.548	4.955	**0.008**
Leaf TN	T changes	117.051	2	58.525	0.717	0.489
	P addition	21.698	1	21.698	0.266	0.607
	T changes *P addition	23.054	2	11.527	0.141	0.868
Leaf TP	T changes	61.672	2	30.836	5.157	**0.007**
	P addition	5.491	1	5.491	0.918	0.253
	T changes *P addition	24.191	2	12.095	2.073	0.079
Leaf C/N ratio	T changes	6.349	2	3.174	0.598	0.669
	P addition	0.045	1	0.045	0.011	0.918
	T changes *P addition	0.280	2	0.140	0.026	0.974
Leaf C/P ratio	T changes	7732.952	2	3866.476	5.154	**0.007**
	P addition	135.756	1	135.756	0.181	0.671
	T changes *P addition	1853.307	2	926.654	1.235	0.293
Leaf N/P ratio	T changes	46.807	2	23.403	6.410	**0.002**
	P addition	0.346	1	0.346	0.095	0.759
	T changes *P addition	16.202	2	8.101	2.219	0.111

Bold numbers indicate significant differences (p < 0.05). *Meant interaction.

### Effect of Warming and P Addition on the C, N, and P Contents and Stoichiometric Characteristics of *Potamogeton crispus* Stems

**FIGURE 2:**
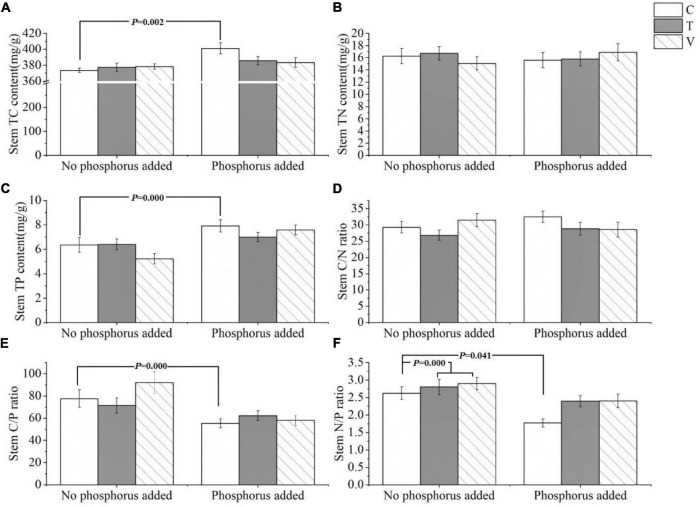
The stoichiometric characteristics of the **(A)** TC, **(B)** TN, and **(C)** TP contents and the **(D)** C/N, **(E)** C/P, and **(F)** N/P ratios of *P. crispus* stems under different treatments. The C in “No phosphorus added” represent controls (C), T in “No phosphorus added” represent constant warming (T), and V in “No phosphorus added” represent variable warming (V). Meanwhile, the C in “Phosphorus Added” represent phosphorus addition (C + P), T + P in “Phosphorus Added” represent constant warming and phosphorus addition (T + P), and V + P in “Phosphorus Added” represent variable warming and phosphorus addition (V + P).

The C (*p* = 0.002) and P (*p* = 0.000) contents and C/P ratio (*p* = 0.000) in the stem of *P. crispus* varied significantly with P addition. Specifically, the C and P contents of the stems increased with P addition, and the C/P ratio decreased with P addition (Table 2 and Figures 2A,C,E), whereas the N content and C/N ratio showed no significant response (*p* > 0.05) (Table 2 and Figures 2B,D). The N/P ratio was significantly affected by warming and P addition, increasing significantly with warming (*p* = 0.041) and decreasing when P was added (*p* = 0.000) (Table 2 and Figure 2F).

**TABLE 2:** Effects of different temperature scenarios and phosphorus addition on the TC, TN, and TP contents and the C/N, C/P, and N/P ratios in *P. crispus* stems.

Parameters	Factors	SS	df	MS	*F*	*p*-value
Stem TC	T changes	1779.633	2	889.816	0.912	0.403
	P addition	10085.212	1	10085.212	10.334	**0.002**
	T changes *P addition	5331.021	2	2665.510	2.731	0.067
Stem TN	T changes	4.549	2	2.275	0.042	0.959
	P addition	0.392	1	0.392	0.007	0.932
	T changes *P addition	81.699	2	40.850	0.753	0.472
Stem TP	T changes	16.423	2	8.212	1.219	0.298
	P addition	109.468	1	109.468	16.250	**0.000**
	T changes *P addition	26.859	2	13.430	1.994	0.139
Stem C/N ratio	T changes	111.827	2	55.913	0.443	0.642
	P addition	135.114	1	135.114	1.071	0.302
	T changes *P addition	192.797	2	96.398	0.764	0.467
Stem C/P ratio	T changes	3109.184	2	1554.592	1.144	0.321
	P addition	23060.584	1	23060.584	16.966	**0.000**
	T changes *P addition	5365.556	2	2682.778	1.974	0.142
Stem N/P ratio	T changes	7.533	2	3.766	3.247	**0.041**
	P addition	16.745	1	16.745	14.436	**0.000**
	T changes *P addition	1.718	2	0.859	0.741	0.478

Bold numbers indicate significant differences (p < 0.05). *Meant interaction.

### Effect of Warming and P Addition on the C, N, P Contents and Stoichiometric Characteristics of *Potamogeton crispus* Turions

**FIGURE 3:**
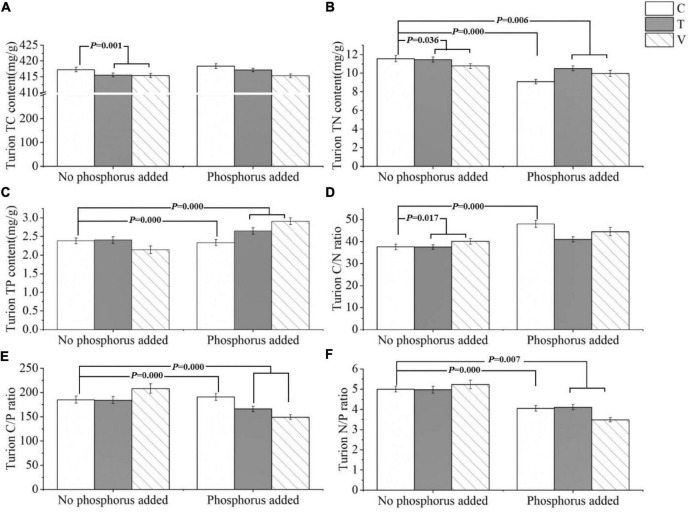
The stoichiometric characteristics of the **(A)** TC, **(B)** TN, and **(C)** TP contents and the **(D)** C/N, **(E)** C/P, and **(F)** N/P ratios of *P. crispus* turions under different treatments. The C in “No phosphorus added” represent controls (C), T in “No phosphorus added” represent constant warming (T), and V in “No phosphorus added” represent variable warming (V). Meanwhile, the C in “Phosphorus Added” represent phosphorus addition (C + P), T + P in “Phosphorus Added” represent constant warming and phosphorus addition (T + P), and V + P in “Phosphorus Added” represent variable warming and phosphorus addition (V + P).

The C content of *P. crispus* turions was significantly affected by warming and when the temperature increased, the C content of *P. crispus* turions decreased significantly (*p* = 0.001) (Table 3 and Figure 3A). N content in the turions was significantly affected by warming (*p* = 0.036), P addition (*p* = 0.000), and their interactive effects (*p* = 0.006). Furthermore, when the temperature increased, the N content of the turions decreased significantly. The N content of the turions also decreased significantly with P addition, warming, and their interactive effects (Table 3 and Figure 3B). Turion P content was affected by P addition, warming (*p* = 0.000), and their interactions (*p* = 0.000). When only P was added, the turion P content decreased, and when P addition and warming interacted, the P content increased (Table 3 and Figure 3C). The turion C/N ratio significantly changed under warming (*p* = 0.017) and P addition (*p* = 0.000), both of which significantly increased (Table 3 and Figure 3D). Turion C/P and N/P ratios were significantly influenced by P addition (*p* = 0.000 and *p* = 0.000, respectively), and both significantly decreased with the interactive effects of warming and P addition (*p* = 0.000 and *p* = 0.007) (Table 3 and Figures 3E,F).

**TABLE 3:** Effects of different temperature scenarios and phosphorus addition on the TC, TN, and TP contents and the C/N, C/P, and N/P ratios in *P.crispus* turions.

Parameters	Factors	SS	df	MS	*F*	*p*-value
Turion TC	T changes	293.050	2	146.525	7.049	**0.001**
	P addition	56.192	1	56.192	2.703	0.101
	T changes *P addition	33.482	2	16.741	0.805	0.448
Turion TN	T changes	26.264	2	13.132	3.376	**0.036**
	P addition	142.479	1	142.479	36.633	**0.000**
	T changes *P addition	40.789	2	20.395	5.244	**0.006**
Turion TP	T changes	2.030	2	1.015	2.809	0.062
	P addition	6.684	1	6.684	18.501	**0.000**
	T changes *P addition	7.190	2	3.595	9.951	**0.000**
Turion C/N ratio	T changes	785.359	2	392.679	4.126	**0.017**
	P addition	2601.546	1	2601.546	27.333	**0.000**
	T changes *P addition	563.060	2	281.530	2.958	0.054
Turion C/P ratio	T changes	7751.299	2	3875.649	1.651	0.194
	P addition	38866.707	1	38866.707	16.553	**0.000**
	T changes *P addition	50552.946	2	25276.473	10.765	**0.000**
Turion N/P ratio	T changes	1.823	2	0.911	0.804	0.448
	P addition	99.824	1	99.824	88.118	**0.000**
	T changes *P addition	11.538	2	5.769	5.092	**0.007**

Bold numbers indicate significant differences (p < 0.05). *Meant interaction.

### Effect of Warming and P Addition on the C, N, P Contents and Stoichiometric Characteristics of *Potamogeton crispus* Seeds

**FIGURE 4:**
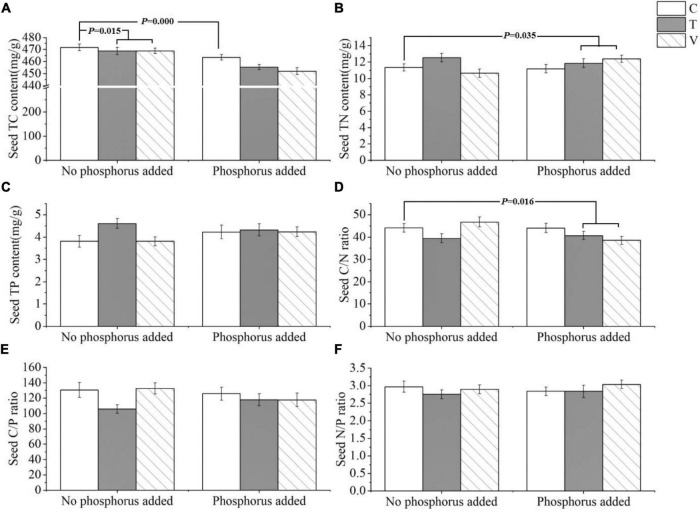
The stoichiometric characteristics of the **(A)** TC, **(B)** TN, and **(C)** TP contents and the **(D)** C/N, **(E)** C/P, and **(F)** N/P ratios of *P. crispus* seeds under different treatments. The C in “No phosphorus added” represent controls (C), T in “No phosphorus added” represent constant warming (T), and V in “No phosphorus added” represent variable warming (V). Meanwhile, the C in “Phosphorus Added” represent phosphorus addition (C + P), T + P in “Phosphorus Added” represent constant warming and phosphorus addition (T + P), and V + P in “Phosphorus Added” represent variable warming and phosphorus addition (V + P).

The *P. crispus* seed C content was significantly affected by warming and P addition, and it decreased with increasing temperatures (*p* = 0.015) and P enrichment (*p* = 0.000) (Table 4 and Figure 4A). The N content (*p* = 0.035) and C/N ratio (*p* = 0.016) were significantly affected by the interaction between warming and P addition. Furthermore, the N content increased under the interaction between warming and P addition, whereas the C/N ratio decreased under this interaction (Table 4 and Figures 4B,D). The P content, C/P ratio, and N/P ratio showed no significant response (Table 4 and Figures 4C,E,F).

**TABLE 4:** Effects of different temperature scenarios and phosphorus addition on the TC, TN, and TP contents and the C/N, C/P, and N/P ratios in *P. crispus* seeds.

Parameters	Factors	SS	df	MS	*F*	*p*-value
Seed TC	T changes	1728.250	2	864.125	4.285	**0.015**
	P addition	7299.843	1	7299.843	36.200	**0.000**
	T changes *P addition	541.646	2	270.823	1.343	0.264
Seed TN	T changes	27.473	2	13.737	1.935	0.148
	P addition	3.864	1	3.864	0.544	0.462
	T changes *P addition	48.644	2	24.322	3.426	**0.035**
Seed TP	T changes	6.111	2	3.056	1.813	0.166
	P addition	0.739	1	0.739	0.438	0.509
	T changes *P addition	3.759	2	1.879	1.115	0.330
Seed C/N ratio	T changes	402.631	2	201.316	1.900	0.153
	P addition	226.661	1	226.661	2.140	0.145
	T changes *P addition	892.274	2	446.137	4.211	**0.016**
Seed C/P ratio	T changes	7959.555	2	3979.778	2.174	0.117
	P addition	286.484	1	286.484	0.156	0.693
	T changes *P addition	5126.994	2	2563.497	1.400	0.250
Seed N/P ratio	T changes	0.404	2	0.202	0.393	0.676
	P addition	0.169	1	0.169	0.329	0.567
	T changes *P addition	0.784	2	0.392	0.762	0.468

Bold numbers indicate significant differences (p < 0.05). *Meant interaction.

## Discussion

### The Response of *Potamogeton crispus* Turions and Seeds to Warming and P Addition

*P. crispus* practices both asexual and sexual reproduction and relies mainly on specialized turions for reproduction (Shen et al., 2008). Many researchers believe that low temperatures and sufficient light are beneficial for the germination of turions, whereas high temperatures inhibit their germination (Jian et al., 2003; Gao et al., 2005; Chen et al., 2006). The C and N contents in turions decreased slightly when the temperature increased, possibly because their growth was inhibited as the temperature increased, which results in a decrease in available inorganic C for turion sequestration. The N content of plants at high temperatures is usually related to respiration (Tjoelker et al., 1999; Crous et al., 2017). The previous studies have shown that an increase in C utilization leads to a decrease in the N content of submerged plants (Madsen et al., 1998; Dülger et al., 2017; Zhang et al., 2020). Our results are consistent with this observation because there is a negative covariance between plant C and N contents (Zhang et al., 2020), which indicates that these plants can self-regulate their internal nutritional composition. However, the N content decreased after P was added, as well as under the combined effects of P input and warming, which was inconsistent with our hypothesis. One possible explanation for this is that algal growth increases as the external nutrient load increases (Strecker et al., 2004; Daufresne et al., 2009) because algae may compete with submerged plants for inorganic C and light (Jones et al., 2002). Nitrogen is the basic component of all enzymes and chlorophyll in plants and plays a key role in controlling carbon assimilation and primary production (LeBauer and Treseder, 2008; Chen et al., 2013). As the C and N contents of the external nutrient load were low, the C and N contents of turions decreased after warming and the addition of P. Regarding the germination conditions of turions, their P content increased after the addition of P, which was consistent with our hypothesis. The addition of P led to a significant increase in the C/N ratio of the turions and a decrease in the C/P and N/P ratios. Under the interactive effects of warming and P enrichment, the C/P and N/P ratios decreased, which indicates that the utilization rate of P increased. The interactive effect of P addition and warming may increase the rate of P utilization by turions. Thus, warming may inhibit the growth of turions.

Carbon provides the foundation for growth, reproduction, and structure (Hessen et al., 2004; Elser et al., 2007). Under phosphorus addition, the decrease in seed C content may be caused by the distribution of more nutrients to the turions. Different C contents may be linked to the germination rate of the propagules. Whereas seeds, as sexual propagules, are important in long-distance transmission, the studies have shown that the germination rate of *P. crispus* seeds is extremely low, only 0.001% (Rogers and Breen, 1980); however, turions, the asexual propagules of *P. crispus*, are widely transplanted in artificial cultivation technology. The decrease in the C content after heating may have been caused by the high temperature. Other studies have demonstrated that temperature controls the germination rate and kinetics of charophytes, and that higher temperatures are not conducive to seed germination (Bonis and Grillas, 2002). Therefore, high temperatures can inhibit seed production. However, after the interaction of P and heating, the N content of the seeds increased and the C/N ratio decreased significantly. This suggests that when both factors act on the seeds simultaneously, the utilization efficiency of N may increase. These changes may further affect the germination rate and average germination time of propagules (Caliskan and Makineci, 2014), thereby affecting the distribution and abundance of the plant’s population. The stoichiometry of seeds was less sensitive to warming and P input and may be more stable than other organs because its function is to maintain the ability to reproduce in response to environmental changes.

### The Response of *Potamogeton crispus* Leaves and Stems to Warming and P Input

*P. crispus* grows poorly under warmer conditions and most of them decay in the summer (Chen, 1985). The optimal temperature range for *P. crispus* is 10–12°C, and temperatures above and below this range affect its growth. Regarding the effects of temperature and nutrients on cold-water plants, the studies have shown that elevated temperatures deplete nutrient elements, thereby limiting the growth of *P. crispus*, and the effects on *P. crispus* stoichiometry are highly dependent on the nutrient conditions of the environment (Zhang et al., 2020). *P. crispus* grows faster in nutrient-rich sediments than in nutrient-poor sediments (Zhang et al., 2019). However, warming and nutrient addition may lead to increases in algae, turbidity, and total suspended matter, which may also inhibit the growth of *P. crispus* (Yan et al., 2021). Global warming and eutrophication can affect the structure and function of aquatic ecosystems by inhibiting the growth of submerged plants, which may lead to the freshwater ecosystem stability becoming more vulnerable in winter and spring. In this experiment, the content of C and P in the leaves decreased significantly with increasing temperature, which indicates that higher temperatures inhibit the growth of *P. crispus* leaves, which is in line with our hypothesis. Plants grown at higher temperatures have a lower respiration rate, which affects their metabolism and leaf growth (Dusenge et al., 2019). During the vigorous growing season, there is a high demand for P to produce sufficient rRNA and synthesize proteins. Therefore, during this period, the P content in the plant increases, which leads to a decrease in the N/P ratio (Gorokhova and Kyle, 2002). The C/P ratio of plant leaves is an important indicator of the physiological metabolism of plants, which can reflect the efficiency of plant P utilization; consequently, fast-growing organisms usually have a lower C/P ratio (Elser et al., 2000; Sterner and Elser, 2017). Our study showed that adding P increased the leaf utilization rate of P, which was reflected in the decrease in the C/P and N/P ratios of the leaves. The temperature-plant physiological hypothesis (Reich and Oleksyn, 2004) suggests that when temperatures rise, plants invest less nutrients into producing the proteins to maintain biochemical reactions (Oleksyn et al., 1998; Tjoelker et al., 1999; Xia et al., 2014), and thus, the C/P and N/P ratios increase significantly. Our results were consistent with this hypothesis. After heating, the C/N ratio of *P. crispus* leaves increased, reflecting the lower N-based biomass per unit C of the plant, which suggests that climate warming improved its nutrient use efficiency. Studies have shown that *P. crispus* is affected by an increase in temperature during the growing season and that the C/N ratio increases. Other angiosperms and phytoplankton communities have also shown a similar temperature-driven increase (Reich and Oleksyn, 2004; Domis et al., 2014; Zhang et al., 2016). However, in this experiment, the leaves were not sensitive to N reactions; therefore, the C/N ratio increased but not significantly. In nature, warming does not have an inhibitory effect on all organisms, and the overall trend indicates that warming is beneficial to smaller biota, such as phytoplankton. It does this by increasing the mineralization of organic carbon and offsetting the direct impact of the increase in the carbon storage capacity of the ecosystem caused by the increase in atmospheric carbon dioxide (Daufresne et al., 2009; Finkel et al., 2010). This may explain why some studies have not detected an effect of rising ambient temperature on the C/N ratio of certain plants.

Submerged plants tend to elongate their stems to reduce the stress associated with low-light conditions (Chen et al., 2016). In this experiment, when nutrient enriched, the C and P contents in the stem of *P. crispus* increased during the elongation stage. Stems play an indispensable role in connecting the aboveground and underground parts of plants. Therefore, *P. crispus* stems may need to meet its internal nutrient transport requirements to resist the stress associated with light reductions (Marschner, 2011; Grasset et al., 2015). This study proves that the addition of P increases the efficiency of the stem. Taken together, the stems also follow the temperature-plant physiology hypothesis (Reich and Oleksyn, 2004), and the N/P ratio increases significantly when the temperature increases. Studies have shown that under moderate nutrient concentrations (TN: 3 mg/L, TP: 0.2 mg/L), warming reduces the stem N/P ratio of *P. crispus* (Yan et al., 2021). However, in this study, the interaction did not have a significant effect on the N/P ratio, which may be caused by different nutrient concentrations. In this experiment, warming led to a reduction in the C and P contents in leaves, but had no significant effect on the C and P contents in stems. Furthermore, warming had less effect on the N content of the leaves and stems. This result indicated that warming was not conducive to the effective utilization of C and P in *P. crispus* leaves, which results in a decrease in the leaves’ ability to accumulate C and P. The leaves of submerged plants may become more heated than the stems because of their larger surface area. In addition, we found that nutrient addition had a greater effect than warming on the stoichiometric characteristics of stems.

## Conclusion

We conclude that different plant organs exhibit different responses to P addition and warming, which demonstrates the importance of assessing the responses of different submerged plant organs to environmental changes. Furthermore, interactive effects between P addition and warming were observed in the leaf, turion, and seed C:N:P stoichiometry, which highlights the importance of multifactorial studies on this topic. Our data indicated that warming resulted in a decrease in the C content in most organs except the stems; P addition increased the P content in most organs except the seeds and turions; and the N content in the seeds were affected by an interactive effect of both conditions. We also found that P addition had a greater effect than warming on the stoichiometric characteristics of the stem. Overall, the addition of P can help *P. crispus* to resist the adverse effects of high temperatures by aiding growth and asexual reproduction, and asexual propagules are more sensitive to P enrichment than sexual propagules.

## Data Availability Statement

The raw data supporting the conclusions of this article will be made available by the authors, without undue reservation.

## Author Contributions

YW, MD, TW, and JX designed the study. TW and MD conducted the field and laboratory measurements. MD and YW analyzed the data and wrote the manuscript. TW, JX, and YW revised the manuscript. All authors have approved the final manuscript.

## Conflict of Interest

The authors declare that the research was conducted in the absence of any commercial or financial relationships that could be construed as a potential conflict of interest.

## Publisher’s Note

All claims expressed in this article are solely those of the authors and do not necessarily represent those of their affiliated organizations, or those of the publisher, the editors and the reviewers. Any product that may be evaluated in this article, or claim that may be made by its manufacturer, is not guaranteed or endorsed by the publisher.
