# Supplementary material for: Effects of Warming and Phosphorus Enrichment on the C:N:P Stoichiometry of Potamogeton crispus Organs
Source: Front Plant Sci. 2022 Mar 29;13:814255. doi: 10.3389/fpls.2022.814255 (PMC9002266; doi:10.3389/fpls.2022.814255)
Supplement: Supplementary file 1 [file Table_1.DOCX]

Supplementary Material

**Table S1** Water chemistry values (mean ± standard error) for the different treatment groups (6 replicates per group) over the duration of the experiment (*n* = 192).

|  | TN (mg/L) | TP (mg/L) | N:P | Chl.*a* (mg/m^3^) |
| --- | --- | --- | --- | --- |
| C | 0.696 ± 0.055 | 0.062 ± 0.007 | 26.530 ± 4.642 | 5.134 ± 0.997 |
| T | 0.847 ± 0.063 | 0.071 ± 0.009 | 26.190 ± 4.321 | 16.452 ± 3.201 |
| V | 0.790 ± 0.055 | 0.069 ± 0.007 | 26.160 ± 3.701 | 12.498 ± 3.464 |
| C+P | 0.724 ± 0.060 | 0.078 ± 0.007 | 17.941 ± 3.996 | 18.078 ± 4.894 |
| T+P | 0.852 ± 0.060 | 0.095 ± 0.010 | 16.530 ± 2.574 | 19.910 ± 4.335 |
| V+P | 0.799 ± 0.066 | 0.084 ± 0.008 | 15.096 ± 2.398 | 14.328 ± 3.178 |

C represent controls; T represent constant warming; V represent variable warming; C+P represent phosphorus addition; T+P represent constant warming and phosphorus addition; V+P represent variable warming and phosphorus addition.
